# The Impact of Excessive Muscle Co‐Contraction on Sit‐To‐Stand Performance in High‐Heeled Footwear

**DOI:** 10.1049/htl2.70011

**Published:** 2025-05-09

**Authors:** Ganesh R. Naik, Amit N. Pujari

**Affiliations:** ^1^ College of Medicine and Public Health Flinders University Adelaide Australia; ^2^ Design and Creative Technology Vertical Torrens University Adelaide Australia; ^3^ Neu(RAL)^2^: NeuRAL Systems & Rehabilitation and Assistive Technologies Laboratory, School of Physics, Engineering and Computer Science University of Hertfordshire Hatfield England; ^4^ School of Engineering University of Aberdeen Aberdeen Scotland

**Keywords:** co‐contraction, electromyography, High heel shoes, Sit to stand

## Abstract

This study aimed to analyse the effects of co‐contraction on quadriceps and hamstring muscles during sit‐to‐stand (STS) tasks for females wearing shoes with different heel heights. The study aimed to identify compensatory strategies during the STS tasks in response to excessive muscle co‐contraction during high‐heeled gait. Sixteen healthy young women (age: 24.4 ± 1.7 years, body mass index: 18.4 ± 1 kg/m^2^, weight: 50.2 ± 5.2 kg, height: 1.63 ± 4.4 m) participated in this study. Electromyography signals were recorded from three quadriceps (vastus medialis, vastus lateralis, and rectus femoris) and one hamstring (semitendinosus) muscles. The participants wore shoes with different heights, including 4, 6, 8, and 10 cm. For each heel height, the co‐contraction index is computed to measure postural balance using the quadriceps to hamstring muscle pairs. The results that were obtained and quantified with statistical measures show that for elevated shoes, if co‐contraction increases, both quadriceps and hamstring muscles tend to compensate. This suggests that the capacity of the quadriceps and hamstring muscles to compensate is essential to retain normal walking and STS tasks in co‐contracted persons. However, the compensation mechanisms may induce imbalance, muscle stiffness, and fatigue for women who regularly use high‐heeled shoes during sit‐to‐stand tasks.

## Graphical abstract

This exploratory study aimed to quantify the effect of co‐contraction for high heeled shoes (HHS). The results support the hypothesis that quadriceps to hamstring co‐contraction increases for elevated HHS. Our study findings indicated that capacity of the quadriceps and hamstring muscles to compensate is fundamental for retaining normal sit to stand (STS) tasks with muscle co‐contraction. From the results, it could be expected that elevated HHS exerts more external work to maintain the same quadriceps‐to‐hamstring ratio compared to lower HHS. Hence, the compensation mechanisms used by lower limb muscles may induce imbalance, muscle stiffness, and fatigue with regular usage of high heel shoes in women during the STS task.

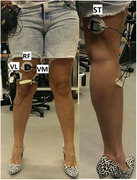

## 1 Introduction

Nowadays, women regularly wear high‐heeled shoes (HHS) for day‐to‐day activities, which include walking, stair ascent, stair descent, sit‐to‐stand (STS), and stand‐to‐sit‐returning (STSR) tasks. Regular use of HHS has been reported to negatively impact different body structures, change gait mechanics, and result in musculoskeletal problems [1, 2]. STS and STSR tasks are the most frequently performed activities in daily life [3, 4]. These tasks are described as a motion of the human body from a stable sitting‐down position to a straight‐up‐standing position and vice versa [5, 6]. These tasks require higher muscle power and coordination in the balance system than other daily tasks, such as walking and stair climbing [7, 8]. This demands posture adjustments and optimal neuromuscular coordination of the quadriceps and hamstring muscles [3, 9].

Co‐contraction, the synchronized activation of agonist and antagonist muscles (antagonistic pairs), occurs in several daily events, including postural control, walking, and running [10, 11, 12]. Busse et al. [13] report that co‐contraction is the mechanism that regulates the simultaneous activity of agonist and antagonist muscles crossing the same joint. Other research shows that excessive co‐contraction can cause inefficient or abnormal movements in some neuromuscular pathologies and is even associated with normal aging [14, 15]. Excessive or poorly controlled co‐contraction is reported to be a major cause of inefficient gait [16] in individuals with cerebral palsy [17], with potentially negative repercussions on the quality of life [16] such as restricting joint motion and increasing energy expenditure, in individuals with CP [18, 19]. As it has been posited that agonist‐antagonist muscle co‐contraction reflects a deliberate neural control strategy to preserve effector‐level control allowing stabilizing motor actions without having to control individual muscles separately [20]; investigating co‐contraction during regular/daily tasks [10, 11, 12], as well as during specific contractions can provide valuable insights about changing muscle behaviours and neural control strategy [21].

Due to increased mechanical demands associated with STS and STSR tasks, it is reasonable to expect activation of the lower extremity muscles to increase with gait speed [22, 23, 24]. Prior research shows that women appear to preferentially activate the lateral quadriceps and hamstring muscles during STS while simultaneously displaying less medial thigh muscle activation [15]. Moreover, as quoted by Lloyd et al., the quadriceps and hamstring muscles have the potential to provide dynamic knee stability because of their abduction and/or adduction moments [25, 26]. To maintain joint stability and body balance during high loading tasks of STS and STSR, it is crucial that muscle co‐contraction of the involved muscles is optimum, as co‐contraction is known to be used to maintain joint stability, whereas excessive co‐contraction is a result of pathological changes in neuromuscular changes. To our knowledge, the effects of muscle co‐contraction of HHS for STS have not been examined.

Regularly wearing HHS alters the neuromechanics of walking, compromises muscle efficiency, causes discomfort, and increases the risk of strain injuries [1]. Additionally, it has been postulated that HHS may contribute to developing and progressing knee osteoarthritis (OA) [5, 27]. As wearing HHS can not only lead to abnormal alterations in neuromuscular and musculoskeletal behaviour but also contribute to long‐term complications such as osteoarthritis, this study attempts to analyse activation and co‐contraction patterns of quadriceps and hamstring agonist/antagonist muscle pairs to investigate potential neuromuscular alterations caused by HHS. We specifically focus on the rectus femoris (RF), vastus lateralis (VL), vastus medialis (VM) and semitendinosus (ST) during STS of different HHS gait. Two hypotheses were tested in this study. First, we hypothesized that elevated heels would have greater co‐contraction of the quadriceps to hamstring muscles during STS tasks with HHS. The second hypothesis was that elevated heel height would be associated with increased muscle activity in the quadriceps and hamstrings. This may lead to altered joint kinematics and kinetics during the sit‐to‐stand task.

While the ankle joint, stabilised by the anterior tibialis and gastrocnemius muscles, is significantly affected by high‐heeled footwear, this study focused on the knee joint and the co‐contraction of the quadriceps and hamstring muscles. This decision was based on several considerations. Firstly, the knee joint is subjected to increased stress and altered biomechanics due to the changes in weight distribution and joint angles associated with high‐heeled shoes. Secondly, co‐contraction of the quadriceps and hamstrings is a common strategy employed by the body to enhance joint stability and control movement [28, 29]. By analysing the co‐contraction patterns in this muscle pair, we aimed to gain insights into the compensatory mechanisms adopted by individuals to maintain balance and stability during the sit‐to‐stand task while wearing high‐heeled shoes.

## 2 Materials and Methods

An exploratory repeated measures study was conducted using data collected from young female participants for the proposed research. The materials and methods used for this study are explained below.

### 2.1 Participants

The University of Technology Sydney, Sydney (UTS), Human Research Ethics Committee approved the experimental protocol (Ethics details: UTS HREC 2013000728) for this study and adheres to the Declaration of Helsinki. Sixteen healthy young women participated in this study (age: 19.4 ± 1.7 years, body mass index: 21.4 ± 1 kg/m^2^, weight: 50.2 ± 5.2 kg, height: 1.63 ± 0.1 m). As a pilot study, this study was based on a convenience sample (of sixteen women), and no power calculations were done. All the participants had no musculoskeletal disorders or injuries of the lower extremity, were not pregnant, and had no history of surgery on the lower extremity. An information sheet was given, and all participants signed a written consent form in the presence of the researcher before the experiment. The participants were familiar with wearing HHS.

### 2.2 Study Design

**FIGURE 1:**
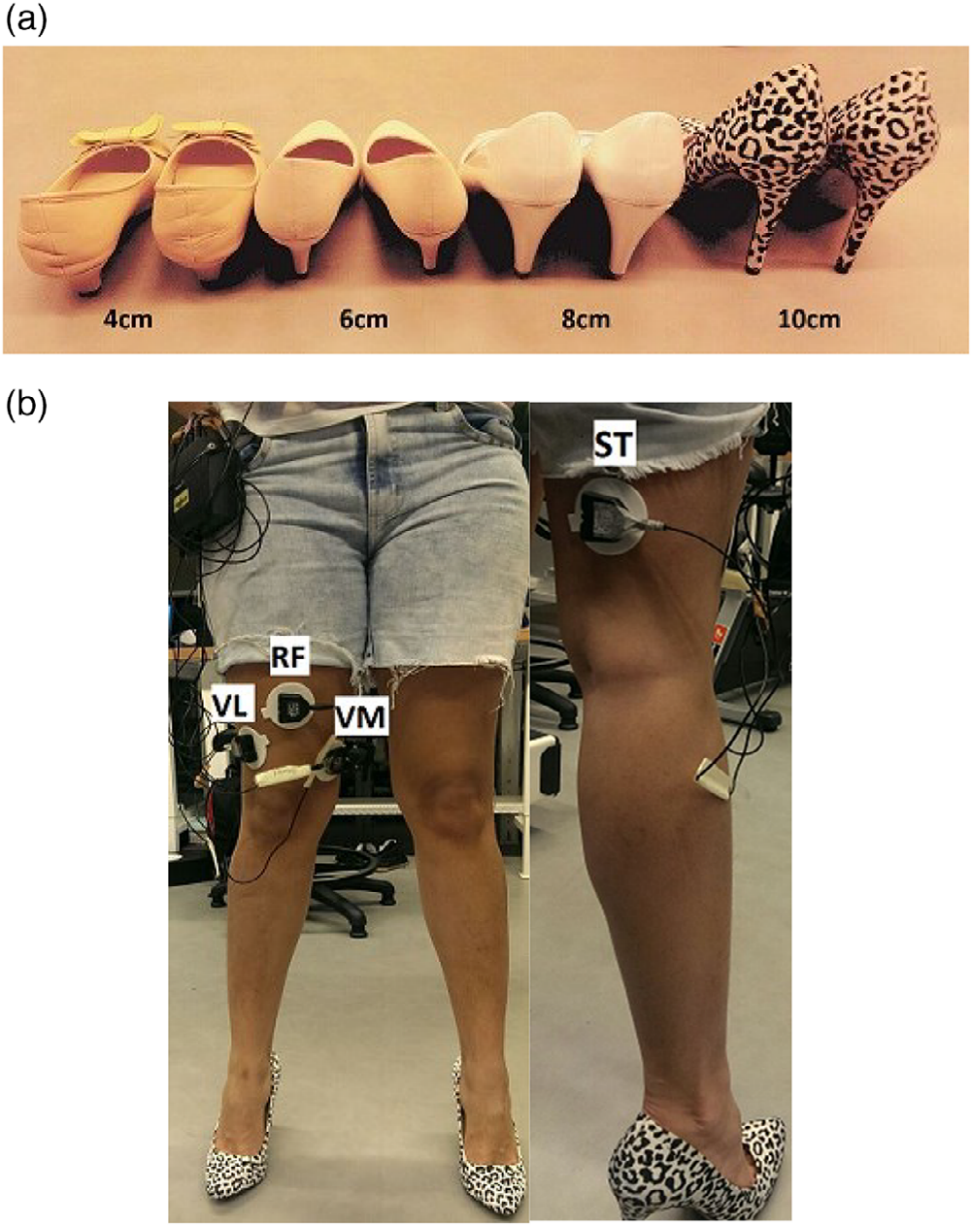
(a) Shoes used for the experiment. (b) Electrode locations for the quadriceps (left) and hamstring (right) muscles.

For this study, shoes with four different heel heights were chosen, including 4, 6, 8, and 10 cm. The shoes used for the experiment are shown in Figure 1a. The surface of the heels is approximately 1 cm^2^ for all shoes, defined as a stiletto in the fashion industry. To maintain control, the shape and style of these shoes are chosen to be as similar as possible.

Participants were not habitual high‐heel wearers. Prior to the experiment, they were introduced to high‐heeled shoes of varying heights. To minimise pre‐experimental muscle strain, participants tried on all shoe heights without weight‐bearing. High‐heeled shoes were worn exclusively during the experimental period.

### 2.3 Data Acquisition

The sEMG signals were recorded from three quadriceps muscles and a hamstring muscle, which include RF, VM, VL, and ST muscles. The quadriceps and hamstring muscle groups, specifically the VM and ST, were selected for this study. These muscles play crucial roles in knee extension and flexion, respectively, and have been extensively studied in relation to lower limb biomechanics and functional tasks such as sit‐to‐stand [30, 31]. While the biceps femoris also contributes to knee flexion, the semitendinosus was chosen due to its larger muscle mass and potential for greater influence on joint moments and power generation during the task [32]. The electrodes were placed on the dominant leg. The electrode locations used for the experiment are shown in Figure 1b. To identify the dominant leg, participants were asked which leg they would choose to kick a ball with, and the chosen leg was considered the dominant one. The placement of electrodes was configured according to the SENIAM guidelines [33]. The electrodes were silver‐silver triode with a fixed inter‐electrode distance of 2 cm and a diameter of 1 cm (Thought Technology, Montreal, Quebec, Canada). Prior to electrode placement, hair was removed from the skin surface, and the skin was cleaned with an alcohol wipe to reduce skin impedance. The skin was then allowed to air dry before electrode placement to ensure optimal signal quality. The sEMG signal was recorded by Flexcomp Infiniti encoder system and transmitted wirelessly to a computer through Bluetooth (Biograph Infiniti) at 2048 samples/s.

All tasks were performed in a health technology laboratory. The sequence of wearing different heights of shoes was randomly assigned. Detailed explanations of the planned experiments were given before data recording so that participants could familiarize themselves with the environment and procedure. For the STS task, participants sat in an armless chair. Participants were instructed to sit on a standard chair with a seat height of 46 cm, with their feet flat on the floor. They were asked to put their arm across their chest. Thus, the arms would not be used to assist the movement of standing up. Their feet were placed in a set position so that movement of feet and legs was not required when they stood up from their seated position. After the participants settled in the chair, they sat still for 5 s and were signalled to stand up by the word ‘stand.’ Participants were required to remain standing for 5 s until they heard the word ‘sit.’ Participants carried out three repetitions of a sit‐to‐stand task under each of four conditions (of wearing 4, 6, 8, and 10 cm high heels). The sit and stand period duration was maintained across all conditions (experiments). After each condition (i.e. after wearing 4 cm HHS, then 6 cm HHS, then 8 cm HHL, etc.), about 3–5 min of rest was given to avoid potential muscle fatigue.

### 2.4 Data Processing

Data were exported from Biograph Infiniti and then processed using MATLAB R2017a software. sEMG data was normalised using the maximal voluntary isometric contraction (MVIC) value (recorded from the same muscle) as the reference value [34]. For that, a sEMG signal from a given muscle was used as the sEMG recorded from the same muscle during MVIC as the reference value. A fourth‐order Butterworth band‐pass filter with a frequency range of 20 to 450 Hz was applied to reject any frequency outside this range, likely to be noise [35, 36]. Due to movement artifacts in the initial and final transient phases of the test, the signals generated during these periods (i.e., before 5% and after 95% of the total time of the test) were discarded.

#### 2.4.1 Quantification of Co‐Contraction

Muscle co‐contraction was assessed between the quadriceps (RF, VL, and VM) and hamstring (ST) muscle pairs, which include VL‐ST, VM‐ST, and RF‐ST combinations. The co‐contraction index (CCI) is computed by summing the ratio of the linear‐enveloped EMG multiplied by the sum of the EMG magnitudes for the quadriceps and hamstring muscle pairs, as described by Nelson–Wong et al. [37].

(1) $$\mathrm{CCI}=\sum_{i=1}^{N}\left(\frac{\mathrm{EM}G_{\mathrm{lo}w_{i}}}{\mathrm{EM}G_{\mathrm{hig}h_{i}}}\right)\left(\mathrm{EM}G_{\mathrm{lo}w_{i}}+\mathrm{EM}G_{\mathrm{hig}h_{i}}\right)$$

where *N* is the total number of data points for the time frame of interest, $\mathrm{EM}G_{\mathrm{low}i}$ is the lower EMG value at the ith data point, and $\mathrm{EM}G_{\mathrm{high}i}$ is the higher EMG value at the ith data point. The CCI provided a measure of the relative activation of the muscle pairs at each instance of the gait cycle [38, 39]. Also, larger and smaller CCI values represent greater and lower muscle co‐contractions, respectively [40]. During the analysis, Equation (1) was applied for the sEMG data of each muscle pair (VL‐ST, VM‐ST, and RF‐ST) that was recorded for a 5 s duration. Since sEMG data were sampled at 2048 Hz, there were 10,240 data points included in each 5‐s window (*N*). For each *i*th point within the 5 s window, the linear‐enveloped EMG magnitudes were compared by taking the low over the high value ratio and then multiplied by the sum of the two magnitudes (Equation (1)). These products were then summed over the 10,240 data points comprising the 5‐s window. The resulting values for 10,240 data points are averaged to produce a single value representing the co‐contraction index for each muscle pair during one STS task. The overall results for the three STS trials were then averaged for statistical analysis. The above procedure was repeated for all HHS tasks and the 16 subjects.

To assess the normality of the data, the Shapiro–Wilk test was performed, and as the data was found to be normally distributed, parametric statistical tests were employed for analysis. A repeated measures analysis of variance (ANOVA) was conducted using MATLAB software to compare the CCI calculated for three muscle pairs (RF‐ST, VM‐ST, and VL‐ST) across four different heel heights, with a statistical significance level set at *p* < 0.05 (95% confidence intervals). Where significant main effects were identified in the repeated measures ANOVA, post‐hoc pairwise comparisons with Bonferroni correction were performed to determine specific differences between heel height conditions and between muscle pairs. All co‐contraction parameters' effect size (small, medium, or large) was assessed using Cohen's *d* (standardized mean differences). Taking into account the cut‐off established by Cohen, the effect size can be small (∼0.2), medium (∼0.5) or large (∼0.8) [41].

## 3 Results

**FIGURE 2:**
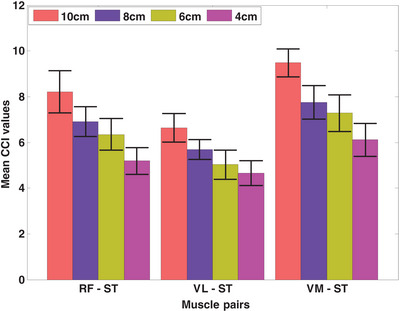
The mean and standard deviation of co‐contraction index (CCI) from the four heel heights for three muscle group combinations. RF, rectus femoris; ST: semitendinosus; VL, vastus lateralis; VM, vastus medialis.

Pooled (mean and standard deviation) CCI from the four heel heights for the three muscle group combinations is shown in Figure 2. The highest CCI ratio was found for the VM‐ST muscle pairs, while the lowest values were found for RF‐ST and VL‐ST variants. The results indicated that the CCI ratios increased for elevated HHS because both quadriceps and hamstring muscles exert higher abduction and/or adduction moments for high‐level muscle activities [15, 26]. Also, due to increased mechanical demands associated with STS tasks, it is reasonable to expect activation of the lower extremity muscles, especially quadriceps and hamstring muscles, to increase with HHS [42]. Predominantly, the simultaneous recruitment of muscles that produce moments in opposite directions, as happens during increased antagonistic muscle co‐contraction around the joint, has a substantial influence on the movement patterns of the knee during STS tasks [43].

**FIGURE 3:**
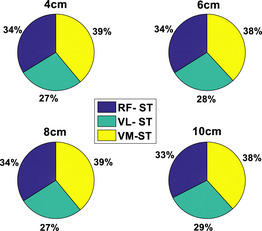
The CCI distribution (%) for RF‐ST, VL‐ST, and VM‐ST for all heel heights (pie‐chart). RF, rectus femoris; ST, semitendinosus; VL, vastus lateralis; VM: vastus medialis.

The distributions of muscle involvement (quadriceps and hamstring) in the different HHS‐STS tasks, as measured by the CCI ratio (percentage of each muscle pair), are shown in Figure 3 in the form of pie chart. From the results, it is interesting that RF‐ST, VL‐ST, and VM‐ST showed similar CCI distribution (approximately 34%, 28%, and 38%, respectively) irrespective of heel height. In other words, the CCI distribution remains constant for RF‐ST, VL‐ST, and VM‐ST for all HHS. This is very likely because both the quadriceps and hamstring muscles have the potential to provide dynamic frontal‐plane knee stability and have the capacity to balance variable abduction‐adduction loads [15, 26].

**FIGURE 4:**
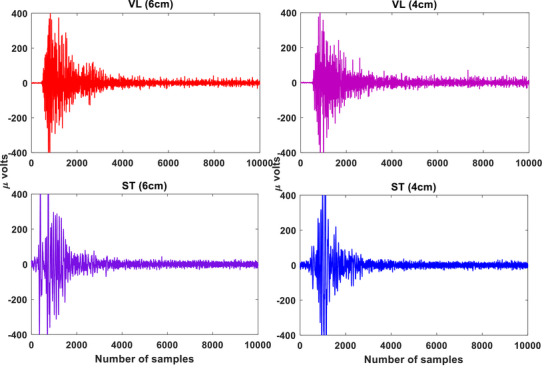
The raw sEMG patterns for VL and ST muscles for 6 and 8 cm heel heights. Here, the *x*‐axis represents the samples and the *y*‐axis represents the amplitude in micro (μ) volts. ST, semitendinosus; VL, vastus lateralis.

**FIGURE 5:**
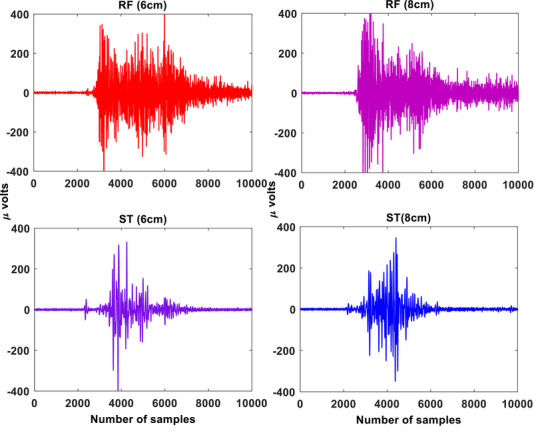
The raw sEMG patterns for RF and ST muscles for 6 and 8 cm heel heights. Here, the *x*‐axis represents the samples and *y*‐axis represents the amplitude in micro (μ) volts. RF, rectus femoris; ST, semitendinosus.

**FIGURE 6:**
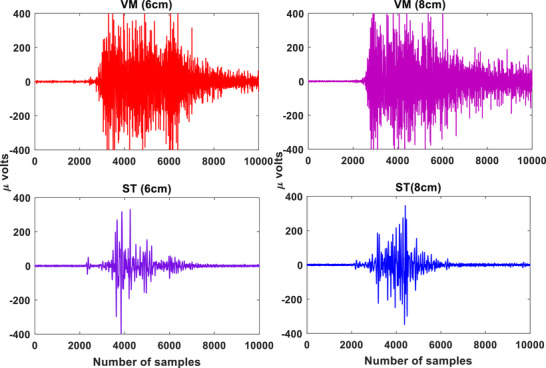
The raw sEMG patterns for VM and ST muscles for 6 and 8 cm heel heights. Here, the *x*‐axis represents the samples and *y*‐axis represents the amplitude in micro (μ) volts. ST, semitendinosus; VM, vastus medialis.

For the RF‐ST co‐contraction, no significant differences were found between 8 and 6 cm HHS (*p* > 0.05; Cohen's *d* = 0.84), as shown in Table 1. The RF‐ST co‐contraction index was highest for 10 cm HHS and lowest for 4 cm HHS. There were significant differences in CCI values between 10–8 cm, 10–6 cm, 10–4 cm, 8–4 cm, and 6–4 cm groups for RF‐ST co‐contraction during STS tasks (*p* < 0.05). For the VL‐ST co‐contraction, no significant differences were found between 6 and 4 cm (*p *> 0.05; Cohen's *d* = 0.64) HHS (Table 2). The VL‐ST co‐contraction index was highest for 10 cm HHS and lowest for 4 cm HHS. Significant differences in CCI values were found between 10–8 cm, 10–6 cm, 10–4 cm, 8–6 cm, and 8–4 cm groups for VL‐ST co‐contraction during STS tasks (*p* < 0.05). For the VM‐ST co‐contraction (similar to RF‐ST), no significant differences were found between 8 and 6 cm HHS (*p *> 0.05; Cohen's *d* = 0.62), as shown in Table 3. The VM‐ST co‐contraction index was highest for 10 cm HHS and lowest for 4 cm HHS. There were significant differences in CCI values between 10–8 cm, 10–6 cm, 10–4 cm, 8–4 cm, and 6–4 cm groups for VL‐ST co‐contraction during STS tasks (*p* < 0.05). The sEMG signal patterns for the VL‐ST, RF‐ST, and VM‐ST for one of the subjects are shown in Figures 4, 5, 6, respectively. Comparable sEMG patterns (Figures 4, 5, 6) were observed for all the participants. From the plots, it is evident that a similar trend of sEMG patterns can be seen for the RF‐ST, 6–8 cm; VL‐ST, 4–6 cm, and VM‐ST, 6–8 cm results. The results show that the VL‐ST pair (refer to Figure 4) exhibits similar properties for 4–6 cm heel heights, whereas RF‐ST and VM‐ST pairs (refer to Figures 5 and 6) show similar results for 6–8 cm heel heights. The results are also justified with high Cohen's index values indicating the similarity among the mentioned HHS pairs. Similar findings are seen in Remaud et al. [44], which show similar muscle activities for VM and RF muscles, whereas specific muscle activity was observed for VL muscle for isotonic and isokinetic contractions.

**TABLE 1:** Pairwise ANOVA (*p*—values) and Cohen's *d* effect size for all four heel heights using RF‐ST.

Heels	8 cm	6 cm	4 cm
10 cm	0.002***, 1.64	0.004***, 2.3	0.004***, 3.9
8 cm		0.348*, 0.84	0.004***, 2.79
6 cm			0.004***, 1.83

*Note*: Significant heel height‐associated differences are indicated by *p > 0.05; ***p < 0.01.

**TABLE 2:** Pairwise ANOVA (*p*—values) and Cohen's *d* effect size for all four heel heights using VL‐ST.

Heels	8 cm	6 cm	4 cm
10 cm	0.0016***, 1.77	0.004***, 2.53	0.003***, 3.41
8 cm		0.039*, 1.21	0.002***, 2.17
6 cm			0.427*, 0.64

*Note*: Significant heel height‐associated differences are indicated by *p > 0.05; *** *p* < 0.01.

**TABLE 3:** Pairwise ANOVA (*p*—values) and Cohen's *d* effect size for all four heel heights using VM‐ST.

Heels	8 cm	6 cm	4 cm
10 cm	0.003***, 2.53	0.002***, 3.11	0.003***, 5.03
8 cm		0.425*, 0.62	0.003***, 2.24
6 cm			0.0032***, 1.53

*Note*: Significant heel height‐associated differences are indicated by *p > 0.05; *** *p* < 0.01.

## 4 Discussion

The major findings of this study are that: (1) irrespective of subjects, in 6–8 cm heel height, compensation (or adjustment) needed to maintain balance or stability between antagonistic muscle pairs RF and ST occur similarly with different co‐contraction values. (2) For 6–8 cm heel height, compensation (or adjustment) to maintain balance or stability between antagonistic muscle pairs VM and ST occurs similarly with different co‐contraction values. (3) For 4–6 cm heel height, compensation (or adjustment) to maintain balance or stability between antagonistic muscle pairs VL and ST occurs similarly with different co‐contraction values.

Understanding how quadriceps and hamstring muscles behave during STS can help clarify the motor control strategies exerted (during STS tasks) using muscle activation patterns to overcome excessive muscle co‐contraction. Goulart and Valls‐Solé [45] reported the differential action of related muscles during the STS task and determined that the pattern of muscle activity remained constant when the initially seated posture changed (participants seated in different places). As stated by Seyedali et al. [42] co‐contractions may represent a limb stiffening strategy to enhance stability during phases of initial heel strike for HHS, which may result in increasing CCI values for elevated HHS.

The CCI of the quadriceps to hamstring ratio remained the same for all four heel heights (Figure 3). Hypothetically, the net effect of the contribution provided by both quadriceps and hamstring muscles should be approximately constant under different co‐contraction levels since the CCI ratio remains the same for all HHS heights. Hence, it could be expected that elevated HHS exerts more external work to maintain the same quadriceps to hamstring ratio compared to lower HHS. Mainly, elevated HHS movements theoretically require the muscle to perform more work on a given STS compared to lower HHS movements. Moreover, if co‐contraction increases, both quadriceps and hamstring muscles can compensate for elevated shoes. The above results are also in agreement with the previous study by Wang and Gutierrez–Farewik [11], which states that due to muscle redundancy, various neuro‐motor strategies may exist to compensate for excessive muscle co‐contraction.

STS tasks demand complex and optimum neuromuscular coordination and postural changes to control the body and prevent loss of balance [22, 46]. According to Dehail et al., [4] the human body must make necessary adjustments to maintain postural balance. One such scenario related to the STS task, where significant modifications or essential adjustments are needed, is wearing high‐heeled shoes. Barton et al., [47] reported that regular usage of high‐heeled shoes for STS and related tasks might contribute to changes in body posture and may induce low back pain in women.

Our research results indicated that the capacity of the quadriceps and hamstring muscles to compensate is fundamental for retaining normal STS tasks with higher muscle co‐contraction for elevated HHS. However, we can argue that women appear to co‐contract their muscles to enhance stability and support during STS tasks with HHS. Women may employ co‐contraction strategies during STS tasks to stabilize and provide extra shock absorption during heel strikes.

The increased muscle co‐contraction observed in this study during the sit‐to‐stand task in high‐heeled footwear suggests that individuals may employ compensatory strategies to maintain balance and stability. High heels significantly alter the body's centre of mass and the distribution of weight on the feet, leading to increased stress on joints, particularly the knees and ankles. To mitigate these effects, individuals may increase co‐contraction of agonist and antagonist muscles around the knee and ankle joints, thereby enhancing joint stability and shock absorption, especially during heel strikes. Furthermore, the reduced ankle dorsiflexion imposed by high heels may necessitate increased muscle activity to maintain balance and propulsion [1, 48].

### 4.1 Theoretical Contribution

This study contributes to the growing research on the biomechanical effects of high‐heeled footwear. By demonstrating increased muscle co‐contraction during the sit‐to‐stand task, this research provides further evidence for the compensatory strategies employed by individuals to maintain balance and stability in challenging footwear conditions. These findings offer valuable insights into the neuromuscular mechanisms underlying gait adaptation and the potential impact of high‐heeled footwear on musculoskeletal health.

### 4.2 Practical Contribution

The findings of this study have practical implications for healthcare professionals, footwear designers, and individuals who frequently wear high‐heeled shoes. Healthcare providers can use this information to educate patients about the potential risks associated with high‐heeled wear, such as increased muscle strain and joint stress. Footwear designers can leverage these findings to develop footwear that minimizes the negative biomechanical effects of high heels, such as by incorporating innovative designs that promote better foot alignment and shock absorption. Individuals who frequently wear high heels can benefit from understanding the potential consequences and consider limiting their use or choosing footwear with lower heels to reduce the risk of musculoskeletal injuries.

### 4.3 Limitations

While this study provides valuable insights into the biomechanical effects of high‐heeled footwear, it is important to acknowledge certain limitations. Firstly, the sample size was relatively small, which may have limited the statistical power of the study. Future research with larger sample sizes can provide more robust evidence. Secondly, the study focused on a specific population of healthy young women, and the results may not be generalizable to other populations, such as older adults or individuals with specific musculoskeletal conditions. Future studies should consider a more diverse range of participants to assess the impact of high‐heeled footwear on different populations.

Future research could also explore the long‐term effects of high‐heeled wear on musculoskeletal health, including the development of chronic pain conditions such as plantar fasciitis and osteoarthritis. Additionally, investigating the impact of different heel heights and shoe styles on muscle activation patterns and joint loading would provide further insights into the mechanisms underlying the adverse effects of high‐heeled footwear. By addressing these limitations and exploring these research directions, we can gain a more comprehensive understanding of the biomechanical consequences of high‐heel wear and develop strategies to mitigate the associated risks.

## 5 Conclusion

The findings of this study have revealed significant differences in quadriceps—hamstring muscle co‐contraction for HHS during STS tasks. Additionally, the lower and higher heel shoes had significant differences in co‐contraction levels of the quadriceps and hamstring musculature. The occurrence of co‐contractions depends on the phase of movements, along with the demands and characteristics of the muscle during STS tasks.

This exploratory study aimed to quantify the effect of co‐contraction for HHS. The results support the hypothesis that quadriceps to hamstring co‐contraction increases for elevated HHS. Our study findings indicated that the capacity of the quadriceps and hamstring muscles to compensate is fundamental for retaining normal STS tasks with muscle co‐contraction. From the results, it could be expected that elevated HHS exerts more external work to maintain the same quadriceps‐to‐hamstring ratio compared to lower HHS. Hence, the compensation mechanisms used by lower limb muscles may induce imbalance, muscle stiffness, and fatigue with regular usage of high‐heeled shoes in women during the STS task.

There are some inherent limitations in this study. The STS task conditions may have been too similar to reveal differences in co‐contraction. Future efforts should examine the effect of these factors on residual limb activation and co‐contraction patterns.

## Author Contributions

G.R.N. conceptualized the study and completed experiments/data collection. G.R.N. and A.N.P. completed data signal processing and analysis (validation, visualisation). G.R.N. prepared the original draft, and A.N.P. carried out the review and editing. Both G.R.N. and A.N.P. prepared and agreed upon the final version of the manuscript.

## Conflicts of Interest

The authors declare no conflicts of interest.

## Data Availability

All data underlying the findings will be available from the authors upon request.
